# Supplementary material for: Insights into the Novel FAD2 Gene Regulating Oleic Acid Accumulation in Peanut Seeds with Different Maturity
Source: Genes (Basel). 2022 Nov 9;13(11):2076. doi: 10.3390/genes13112076 (PMC9691258; doi:10.3390/genes13112076)
Supplement: Supplementary file 1 [file genes-13-02076-s001.zip › Supplemental tables.pdf]

Table S1 Primers for gene cloning, and qRT-PCR

primer name	ID of PeanutBase	Forward primer sequence （5'-3'）	Forward primer sequence （5'-3'）
FAD2-7	*Arahy.BY45PL*	ATGGGAGCTGGCGGCCGAA	ACTTGGACAGCAGCTCTAGTC
FAD2-8	*Arahy.S1Y1PZ*	ATGGGGTCTGGTGGTCATTCT	CCTCACACTTAAAGCTTATTATACC
FAD2-9	*Arahy.9P5B67*	ATGGGGTCTGGGGGTCATTC	CACTTAAAGCTTATTATACCAAAGA
AhFAD2A/B	*Arahy.42CZAS.1/ Arahy.5913QL.1*	GATTGAAGCTCAAAAGAAGCCTCT	TGTAGAAGAGTAAGTAGGCCACT
AhFAD2-3		ATGCATGCTTTCTTGTCAATTGAAG	AGAGAACGTTCAAAGCAATGTGGT
AhFAD2-7	*Arahy.BY45PL*	AACCTCTGTTCCTCCTTCAAACA	ATAGTAGAGGCAAAAGGCTATGGT
AhFAD2-8	*Arahy.S1Y1PZ*	CATCACACTATCAGTTACTCTCTTT	ATAGAACGCGGCCAGGACAC
AhFAD2-9	*Arahy.9P5B67*	CTCTGGAGAGAGGCCAAAGA	AGCCAACTACGATATATAGCGAT

Table S2 Protein information of new members of FAD2

Gene	ORF（bp）	Chromesome	Lenth of Protein/aa	MW/kDa	pI	Location
AhFAD2-7	1152	B06	383	43.8	8.8	Endoplasmic reticulum
AhFAD2-8	1164	A09	387	45.0	8.96	Endoplasmic reticulum
AhFAD2-9	1164	B09	387	45.1	9.04	Endoplasmic reticulum

Table S3 Fatty acid composition of transformed INVSc1

Transformant	Oleic acid（%）	Linoleic acid（%）
pYES2	12.87	1.70
pYFAD2-7	13.42	10.44
pYFAD2-8	47.22	49.14
pYFAD2-9	55.79	57.03

Table S4 Determination of fatty acids in transgenic Arabidopsis

	Oleic acid（%）	Linoleic acid（%）
WT	11.63	31.01
fad2	57.28	2.56
AhFAD2-7	44.23	7.49
AhFAD2-8	51.45	3.82
AhFAD2-9	39.60	13.06
