# Supplementary figures and images for: Insights into the Novel FAD2 Gene Regulating Oleic Acid Accumulation in Peanut Seeds with Different Maturity
Source: Genes (Basel). 2022 Nov 9;13(11):2076. doi: 10.3390/genes13112076 (PMC9691258; doi:10.3390/genes13112076)

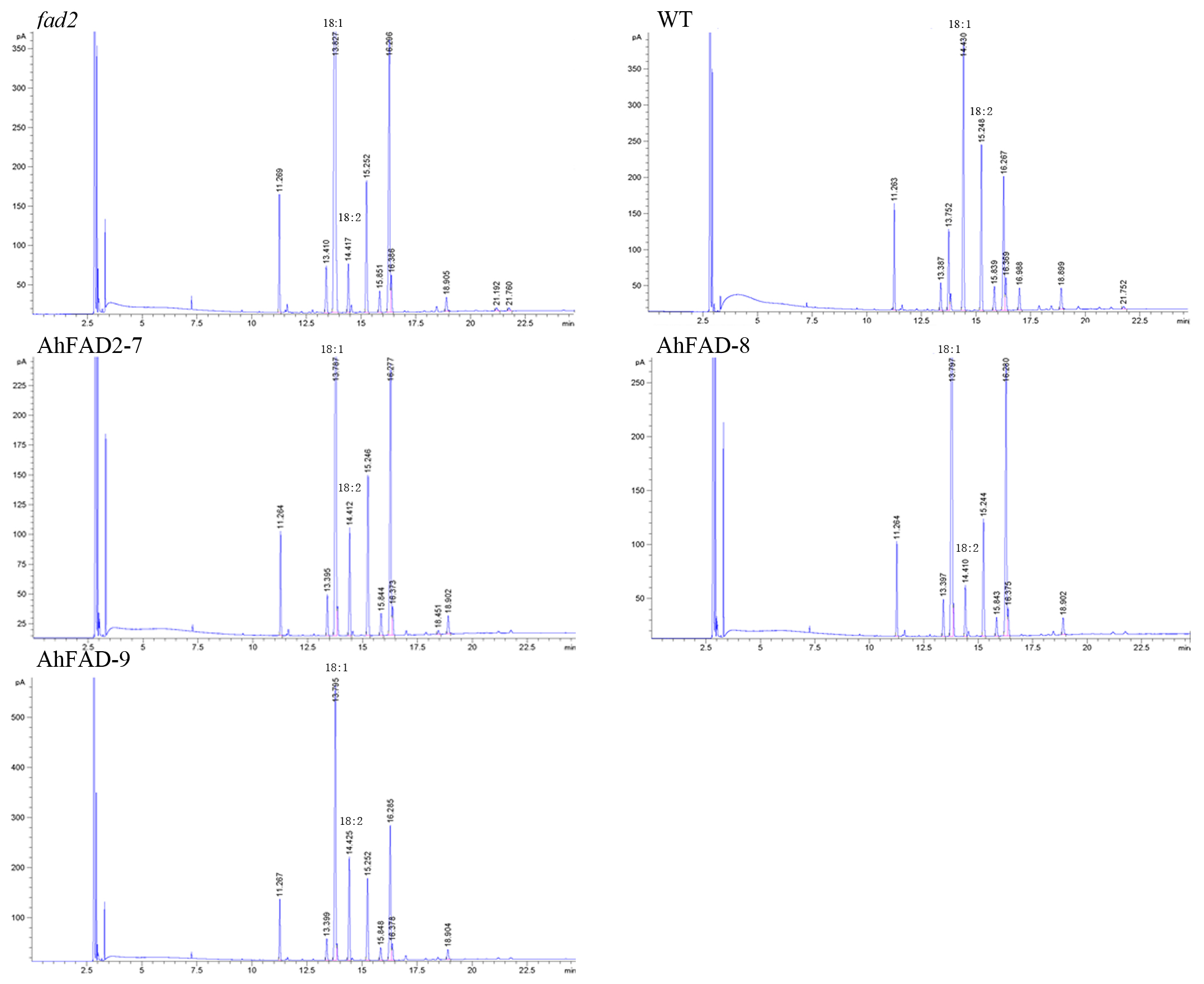

Supplement: Supplementary file 1 [file genes-13-02076-s001.zip › Figure S1.tif]
